# A Facile Synthesis of Nitrogen-Doped Highly Porous Carbon Nanoplatelets: Efficient Catalysts for Oxygen Electroreduction

**DOI:** 10.1038/srep43366

**Published:** 2017-02-27

**Authors:** Yaqing Zhang, Xianlei Zhang, Xiuxiu Ma, Wenhui Guo, Chunchi Wang, Tewodros Asefa, Xingquan He

**Affiliations:** 1 Department of Chemistry and Chemical Engineering, Changchun University of Science and Technology, Changchun 130022, P. R. China; 2 Department of Chemistry and Chemical Biology & Department of Chemical and Biochemical Engineering, Rutgers, The State University of New Jersey, Piscataway, NJ 08854, United States

## Abstract

The oxygen reduction reaction (ORR) is of great importance for various renewable energy conversion technologies such as fuel cells and metal-air batteries. Heteroatom-doped carbon nanomaterials have proven to be robust metal-free electrocatalysts for ORR in the above-mentioned energy devices. Herein, we demonstrate the synthesis of novel highly porous N-doped carbon nanoplatelets (N-HPCNPs) derived from oatmeal (or a biological material) and we show the materials’ high-efficiency as electrocatalyst for ORR. The obtained N-HPCNPs hybrid materials exhibit superior electrocatalytic activities towards ORR, besides excellent stability and good methanol tolerance in both basic and acidic electrolytes. The unique nanoarchitectures with rich micropores and mesopores, as well as the high surface area-to-volume ratios, present in the materials significantly increase the density of accessible catalytically active sites in them and facilitate the transport of electrons and electrolyte within the materials. Consequently, the N-HPCNPs catalysts hold a great potential to serve as low-cost and highly efficient cathode materials in direct methanol fuel cells (DMFCs).

In many fuel conversion systems, the cathodic oxygen reduction reaction (ORR) is deemed a critical process that dictates the efficiency of the chemical energy to electrical energy conversions^1^. The noble metal platinum (Pt) is usually recognized as the most efficient catalyst for ORR because it requires the lowest overpotential and gives the highest current output while catalyzing the desirable 4-electron reduction of dioxygen into water^2^. However, because of its expensiveness, disappointing electron transfer kinetics, and limited supply, platinum-based materials cannot find widespread uses as catalyst for ORR^3^,^4^. Besides, there are also other demerits of Pt-based catalysts, such as their poor tolerance to carbon monoxide (CO) poisoning and methanol crossover effect, and even corrosion, oxidation and deactivation, as well as high tendency to aggregate when it is made with nanoscale structures^5^,^6^. Consequently, it is necessary to explore and find alternative materials to replace platinum-based catalysts that possess good durability, have low cost and show excellent activity for ORR.

Recently, increasing attention has been paid to synthetic methods that can dope heteroatoms (N, B, P, etc.) into carbon matrices to modify the physical, chemical, and electrocatalytic properties of the carbon materials^7^,^8^,^9^,^10^,^11^. Among these materials, N-doped carbon materials have been found to show impressive electrocatalytic properties; they have thus been considered as among the potential substitutes for Pt-based electrocatalysts for ORR^12^,^13^,^14^,^15^,^16^,^17^,^18^,^19^,^20^. It is believed that these materials show such unprecedented catalytic activities due to their unique electronic properties induced by electron donation of the nitrogen dopant atoms into the adjacent carbon atoms on their structures. Compared with the metal-based catalysts, which can easily loss their catalytic activity towards ORR due to the instability of their active sites^21^, N-doped carbon materials including N-doped graphene^9^, N-doped nanofibers^15^, N-doped carbon nanotubes^13^, and N-doped porous carbon^14^ are more advantageous in terms of electrocatalytic activity, operational durability, stability against CO and good tolerance against fuel crossover in fuel cells^22^. Hence, they have been widely researched in recent years.

The porosity of the carbon matrix is often critical for the transport of O_2_ and electrolytes during the ORR processes^23^. So, unsurprisingly the porosity of the carbon matrix in N-doped carbon materials is often critical for their catalytic performances. Hence, constructing micropores combined with mesopores or macropores in the carbon structures is one effective strategy to afford a variety of hierarchically porous carbon nanomaterials with lower resistance to electron and mass transfer processes during electrocatalysis^24^,^25^,^26^. In addition, high porosity and nanoarchitectures give rise to more accessible catalytic sites in the materials, dramatically improving their overall electrocatalytic activity^27^. Therefore, hierarchically porous N-doped carbon nanomaterials can constitute promising candidate catalysts that can potentially replace the costly Pt-based ones in various renewable energy systems.

To this end, herein we introduce novel highly porous N-doped carbon nanoplatelets (N-HPCNPs) synthesized from oatmeal that can serve as highly efficient catalysts for ORR in both acid and alkaline media. Oatmeal is a naturally available, inexpensive, green, and fiber-rich biological material, and it is an abundant source of nitrogen and carbon; thus, we had hypothesized that oatmeal could be utilized as an environmentally friendly material and a promising precursor for making the heteroatom-doped carbon ORR electrocatalysts we have reported herein. The N-HPCNPs are prepared by a two-step method including pyrolysis of oatmeal/urea/zinc acetate/ferric trichloride blends and subsequent etching with acidic solution (Fig. 1). In particular, the N-HPCNPs obtained by pyrolyzing the precursor at 900 °C (abbreviated as N-HPCNPs-900) present unique meso-microporous structure and possesses super-high BET surface area (2633 m^2^ g^−1^). These hybrid carbon catalysts display superior electrocatalytic activity for ORR, excellent methanol tolerance and good durability in both alkaline and acid media. Consequently, the materials have a great potential to be utilized as Pt-free catalyst in various fuel cells, *e.g.*, direct methanol fuel cells (DMFCs).

## Results and Discussion

### Synthesis and characterization of structure and composition of N-HPCNPs-900 and the corresponding control (or reference) materials

N-doped highly porous carbon nanoplatelets (N-HPCNPs) are synthesized by a two-step procedure, involving pyrolysis in Ar atmosphere of oatmeal containing urea, zinc acetate and ferric trichloride, followed by etching the carbonized product with acidic solution (see Experimental Section for details). The N-HPCNPs obtained at different pyrolysis temperatures, namely, 700, 800, 900 and 1000 °C, are named as N-HPCNPs-700, N-HPCNPs-800, N-HPCNPs-900 and N-HPCNPs-1000, respectively. The N-HPCNPs obtained with a pyrolysis temperature of 900 °C and not treated with acid etching are labeled as N-HPCNPs-900-b. For comparative studies, the corresponding reference materials were synthesized without using zinc acetate, ferric trichloride or urea under otherwise similar procedure as the one used to produce N-HPCNPs-900 above, and the respective resulting materials are named as N-P1CNPs, N-P2CNPs and PCNPs. Pristine oatmeal was also pyrolyzed by itself at 900 °C in Ar atmosphere, and the resulting control material is denoted as CNPs.

**Figure 2:**
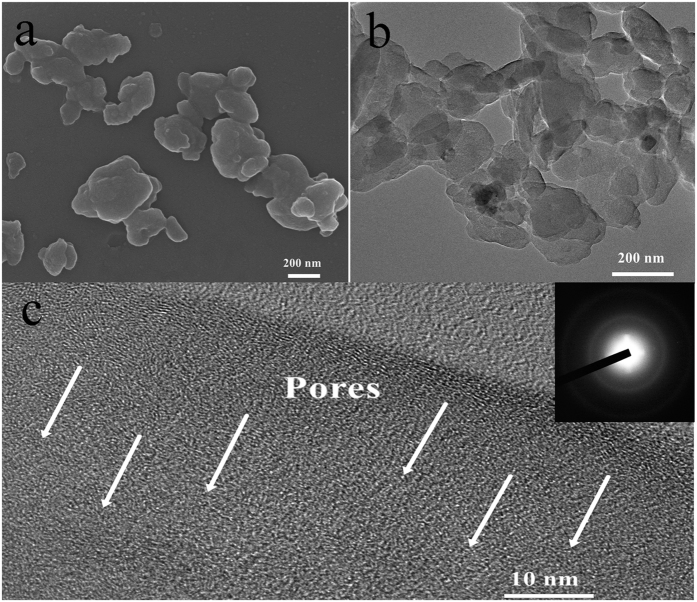
(**a**) Representative scanning electron microscope (SEM) image of N-HPCNPs-900. (**b**) The TEM image of N-HPCNPs-900 and (**c**) the corresponding HRTEM image of N-HPCNPs-900. The inset in (**c**) shows the selected area electron diffraction (SAED) pattern of N-HPCNPs-900.

The morphology of the as-synthesized materials is investigated first with scanning electron microscopy (SEM). The SEM image of N-HPCNPs-900 reveals that the hybrid material has nanoplatelets with irregular morphology and with lateral sizes ranging from several tens to several hundreds of nanometers (Fig. 2a). This is further confirmed by transmission electron microscopy (TEM) (Fig. 2b). The high resolution TEM (HRTEM) image of the N-HPCNPs-900 shows that the carbon plateletes have amorphous structure and micropores with sizes of <2 nm (Fig. 2c). Besides, many dislocation defects can be observed in the graphite layers of the N-HPCNPs-900, most likely due to the structural distortions originated from the incorporation of nitrogen atoms into the graphite lattice of the nanoplatelets. In the inset of Fig. 2c, the selected area electron diffraction (SAED) pattern of the materials displays diffraction rings that are consistent with the typical hexagonal pattern of graphene-like carbon materials possessing poor crystallinity^28^. The Raman spectra of the N-HPCNPs pyrolyzed at different temperatures are shown in Figure S1 in Supplementary Information (SI) section. A typical D band at around 1340 cm^−1^ corresponding to the sp^3^ carbons at defective sites and a G band at 1580 cm^−1^ attributable to the E_2g_ stretching vibration mode of sp^2^ carbons^29^, can be observed in the spectra of all the materials. A low ratio of *I*_D_/*I*_G_ (*i.e*., the intensity of the G band divided by the intensity of the D band) with a value of ~1.0 was obtained for all of the materials, which is indicative of the high degree of graphitization in the N-HPCNPs, possibly due to high pyrolysis temperatures (800–1000 °C) employed to make them^30^.

**Figure 3:**
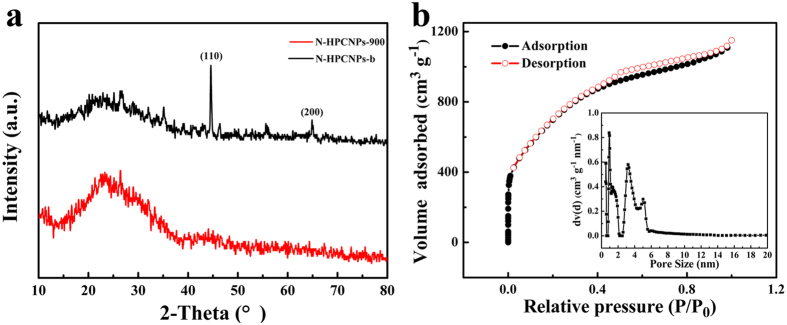
(**a**) XRD patterns of N-HPCNPs-900 and N-HPCNPs-b. (**b**) Nitrogen adsorption-desorption isotherm of N-HPCNPs-900, where the inset shows the pore size distribution of the materials as obtained by the density functional theory (DFT) method.

Figure 3a presents the XRD patterns of N-HPCNPs-900 and N-HPCNPs-b. The XRD patterns of both materials show a sharp diffraction peak at around 24.5°, which can be attributed to the (002) lattice planes of carbon^31^. Besides this, the XRD pattern of N-HPCNPs-b exhibits two strong diffraction peaks at 44.6° and 64.9°, corresponding to the (110) and (200) crystal planes, respectively, of metallic Fe (PDF#41-1487). In the case of the XRD pattern of N-HPCNPs-900, the weak peak observed at around 44.3° is characteristic of graphitic (101) lattice, suggesting the formation of graphitic structure in the materials after pyrolysis. In the XRD pattern of the N-HPCNPs-900, no residual peaks associated with metals are seen, indicating the complete dissolution of Fe during the treatment of the materials with acidic solution.

To further analyze the porosity of the as-obtained N-HPCNPs materials, N_2_ adsorption-desorption measurements were performed (Fig. 3b and Figure S2 in SI). The isotherms of the as-obtained samples show a sharp uptake at low relative pressure (P/P_0_ < 0.015) but a hysteresis loop at high relative pressure (P/P_0_ > 0.45), confirming the existence of abundant micropore and meso/macropore structures in the materials^32^. The Brunauer-Emmett-Teller (BET) surface area of N-HPCNPs-900 is found to be 2633 m^2^ g^−1^, which is much higher than that of CNPs (984 m^2^ g^−1^) (Table 1). The super-high BET surface area of N-HPCNPs-900 is attributed to the nitrogen doping^33^, and the coexistence of ferric trichloride (as a porogen) and zinc acetate (as both porogen and activating agent). What is more, the N-HPCNPs-900 displays a significantly larger pore volume (1.78 cm^3^ g^−1^) than CNPs (0.55 cm^3^ g^−1^). The BET surface area and pore volume of N-HPCNPs-900 are also larger than those of N-HPCNPs-b whose corresponding values are 669 m^2^ g^−1^ and 0.5 cm^3^ g^−1^, respectively (Table 1). This indicates once again that the acid etching is responsible for the formation of large density of pores in the materials. Moreover, the BET surface area of N-HPCNPs-900 is also comparable to, or better than, those of some of the recently reported related porous materials compiled in Table S1 in SI. Obviously, the super-high BET surface area and highly porous architecture of N-HPCNPs-900 can be expected to be conducive for electrocatalysis as they can give rise to more exposed catalytic active sites in the materials and allow better mass transport for reactants and products alike^14^,^34^,^35^,^36^.

**Figure 4:**
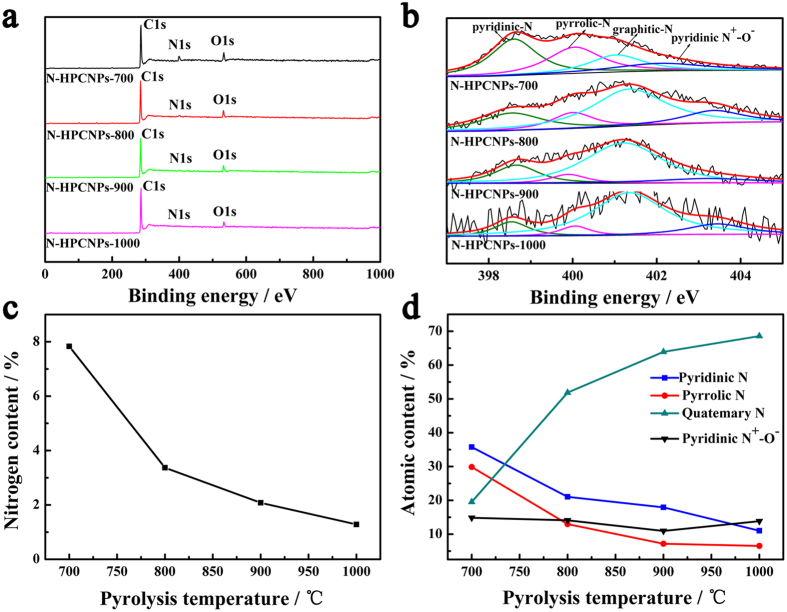
(**a**) XPS survey spectra of N-HPCNPs at different temperatures. (**b**) High-resolution N1s XPS spectra of N-HPCNPs obtained at different pyrolysis temperatures. (**c**) The content of nitrogen in four different N-HPCNPs materials obtained using different pyrolysis temperatures. (**d**) The percentage of four different nitrogen species in nitrogen of N-HPCNPs obtained at different pyrolysis temperatures.

X-ray photoelectron spectroscopy (XPS) measurements are then carried out to analyze the surface compositions as well as the chemical states of the elements in the of N-HPCNPs^37^. As shown in Fig. 4a, the XPS survey spectra of N-HPCNPs obtained with pyrolysis at different temperatures, followed by treatment with acidic solution, show the presence of C, N and O atoms in the materials. However, peaks associated with Fe and Zn are not seen, most likely due to the dissolution of Fe during the etching of the materials with acidic solution and the volatilization of Zn during the pyrolysis process^38^.

To gain better insight into the bonding states in the materials, high-resolution N1s spectra of N-HPCNPs are acquired. The N1s spectra for N-HPCNPs can be fitted into peaks corresponding to pyridinic-N (~398.5 eV), pyrrolic-N (~400.0 eV), graphitic-N (~401.3 eV), and pyridinic N^+^-O^−^ (402.0–404.0 eV) species (Fig. 4b)^21^,^39^,^40^,^41^. The areas under these four peaks are found to significantly vary as with the pyrolysis temperature, indicating the formation of different levels of C, N and O bonding configurations in the materials depending on the pyrolysis temperatures^42^. Specifically, the overall content of N atoms in the materials decreases sharply with the increase in pyrolysis temperature (Fig. 4c). Moreover, as shown in Fig. 4d, increasing pyrolysis temperature leads to the increase of graphitic-N, but the decrease in the amounts of pyridinic-N and pyrrolic-N species, suggesting that high pyrolysis temperature promotes the formation of graphitic-N species in the materials.

### Electrocatalytic measurements

**Figure 5:**
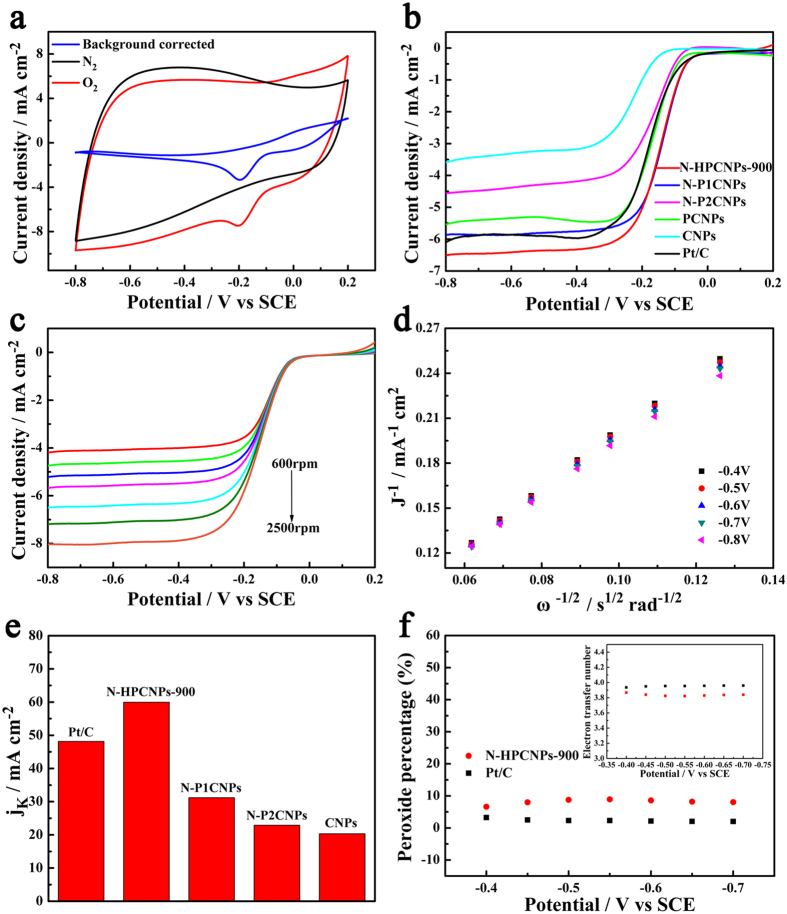
(**a**) CV curves of N-HPCNPs-900 in N_2_- and O_2_-saturated 0.1 M KOH aqueous solutions with a scan rate of 100 mV s^−1^. (**b**) Background-corrected LSV curves of various samples in an O_2_-saturated 0.1 M KOH electrolyte at 1600 rpm with a scan rate of 10 mV s^−1^. (**c**) Background-corrected LSV curves of the N-HPCNPs-900 at different rotating speeds. (**d**) The corresponding Kouteckey-Levich plots of (**c**) at different potentials. (**e**) Summary of the kinetic current density (j_K_) at −0.5 V *vs.* SCE based on the RDE data of various samples. (**f**) Percentage of peroxide and electron transfer number (inset) of N-HPCNPs-900 and Pt/C at fixed potentials of −0.4, −0.45, −0.5, −0.55, −0.6, −0.65 and −0.7 V *vs.* SCE.

The electrocatalytic activity of the materials synthesized above toward ORR was evaluated in both 0.1 M aqueous KOH and 0.5 M aqueous H_2_SO_4_ solutions with cyclic voltammetry (CV) and electrochemical measurements involving rotating disk electrode (RDE) and rotating ring disk electrode (RRDE). In N_2_-saturated 0.1 M aqueous KOH solution, N-HPCNPs-900 show a quasi-rectangular voltammogram with featureless voltammetric current in the potential range from 0.2 to −1.0 V *vs*. SCE (Fig. 5a). In a sharp contrast, when the solution is saturated with O_2_, a pronounced cathodic reduction peak appears at around −0.2 V *vs*. SCE, indicating N-HPCNPs-900’s excellent catalytic activity for ORR. It is worth noting that the N-HPCNPs-900 display a high background non-Faradic current in the CV curve due most likely to N-HPCNPs-900’s large surface area, which can lead to a potential shift in the presence of large background current. As a result, the background-corrected CV (or LSV) curve is obtained by subtracting the CV (or LSV) curve obtained in N_2_-saturated 0.1 M aqueous KOH solution from the corresponding original curve obtained in O_2_-saturated 0.1 M aqueous KOH electrolyte, as presented in Fig. 5a. It can be seen that the N-HPCNPs-900 displays a less negative peak potential in ORR after background correction.

Previous studies have demonstrated that the pyrolysis temperature has a significant effect on the catalytic performances of N-doped carbon materials^43^,^44^. First, the electrocatalytic activity of N-HPCNPs toward ORR as a function of the pyrolysis temperature employed to make them (in the range of 700 to 1000 °C) was evaluated with rotating disk electrode (RDE) in O_2_-saturated aqueous 0.1 M KOH and 0.5 M H_2_SO_4_ solutions. The results display that the N-HPCNPs-900 possesses the highest ORR activity with the most positive onset potential as well as the largest diffusion-limiting current density (Figure S3 in SI).

Background-corrected LSVs of the as-prepared materials are then obtained at a rotation speed of 1600 rpm in an O_2_-saturated 0.1 M KOH solution (Fig. 5b). Some of the electrochemical parameters used for the electrocatalysis of ORR are listed in Table S2 in SI. The N-HPCNPs-900 catalysts exhibit prominent electrocatalytic activity with a half-wave potential of −0.15 V *vs*. SCE and a diffusion-limited current density of 6.50 mA cm^−2^, which are clearly superior to those of the commercial Pt/C catalyst tested under the same conditions (whose half-wave potential is −0.18 V *vs*. SCE and whose current density is 6.09 mA cm^−2^). However, CNPs show clearly weaker catalytic activity toward ORR compared with N-HPCNPs-900. Relative to CNPs, the enhancement in catalytic activity exhibited by N-HPCNPs-900 can be attributed to the N dopants and unique porous scaffolds present in the materials. Moreover, compared with N-P1CNPs or N-P2CNPs, the as-obtained N-HPCNPs-900 shows significantly better electrocatalytic activity toward ORR, which may be attributed to the formation of highly porous structures in the latter due to the combined effects of the two porogens used to make the material.

Figure 5c depicts the background-corrected LSV curves of the N-HPCNPs-900 at different rotation speeds ranging from 600 to 2500 rpm with a scan rate of 10 mV s^−1^. Clearly, the current densities of the catalysts are seen to increase with an increase in the rotation speed of the electrode due to the shortened diffusion distance at higher rotation speeds. The corresponding Koutecky-Levich (K-L) plots of the N-HPCNPs-900 at various rotation speeds are displayed in Fig. 5d. For comparison, the background-corrected LSV polarization curves and the corresponding K-L plots of N-P1CNPs, N-P2CNPs, PCNPs, CNPs and Pt/C are also measured, and the results are depicted in Figure S4 in SI. According to the K-L plots, the kinetic current density (j_K_) of the N-HPCNPs-900 is found to be 59.95 mA cm^−2^ at −0.5 V *vs*. SCE, which is higher than the values obtained for N-P1CNPs (31.20 mA cm^−2^), N-P2CNPs (22.89 mA cm^−2^), PCNPs (50.92 mA cm^−2^) and CNPs (20.31 mA cm^−2^), and even higher than that of the Pt/C catalyst (48.13 mA cm^−2^; see Fig. 5e).

The electrocatalytic activity of N-HPCNPs-900 is also compared with those of other metal-free and non-precious metal-based ORR catalysts reported previously in literature (see Table S3 in SI). The performance of N-HPCNPs-900 is comparable to or even superior to previously reported ORR catalysts in literature, in terms of onset potential, half-wave potential and limiting current density.

In order to quantify the ORR pathways of the N-HPCNPs-900 catalyst, RRDE tests are conducted and the formation of peroxide (H_2_O_2_) during the ORR process is monitored (see Figure S5a in SI). The percentage of the HO_2_^-^ formed over N-HPCNPs-900 is found to be less than 10%, and the corresponding electron transfer number is calculated to be 3.82–3.87 in the potential range from −0.4 to −0.8 V *vs*. SCE. This demonstrates that the reaction almost exclusively involves a four-electron reduction process while giving maximum energy conversion (Fig. 5f).

**Figure 6:**
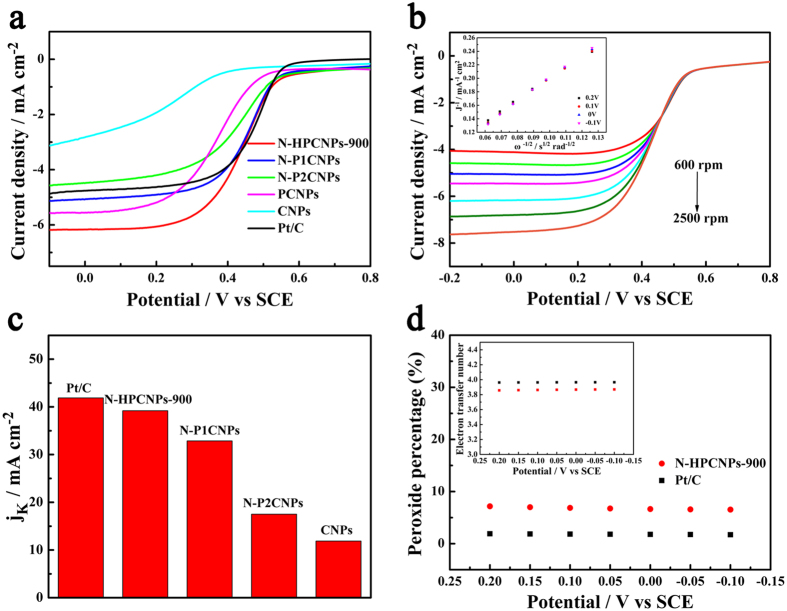
(**a**) Background-corrected LSV curves of various samples in an O_2_-saturated 0.5 M H_2_SO_4_ electrolyte at 1600 rpm with a scan rate of 10 mV s^−1^. (**b**) Background-corrected LSV curves of N-HPCNPs-900 at different rotating speeds and the corresponding Kouteckey-Levich plots (inset) at different potentials. (**c**) Summary of the kinetic current density (j_K_) at −0.1 V *vs.* SCE on the basis of the RDE data on various samples. (**d**) Percentage of peroxide and electron transfer number (inset) of the N-HPCNPs-900 and Pt/C at fixed potentials of 0.2, 0.15, 0.10, 0.05, 0, −0.05 and −0.10 V *vs.* SCE.

N-HPCNPs-900 are also found to exhibit excellent catalytic performances toward ORR in an acidic electrolyte. As shown in Fig. 6a, although the onset potential (0.59 V *vs*. SCE) and half-wave potential (0.45 V *vs*. SCE) of the N-HPCNPs-900 are slightly more negative than those of the Pt/C catalyst (whose onset potential is 0.60 V *vs*. SCE and half-wave potential is 0.47 V *vs*. SCE), the current density of the N-HPCNPs-900 (6.18 mA cm^−2^ at −0.1 V *vs*. SCE) is much higher than that of the commercial Pt/C catalyst at the same catalyst loading (4.87 mA cm^−2^ at −0.1 V *vs*. SCE) (see Table S4 in SI). Background-corrected LSV curves of N-HPCNPs-900 and the corresponding control materials at different rotating speeds are also recorded (Fig. 6b and Figure S6 in SI). The corresponding K-L plots of these hybrid materials are shown in the insets of Fig. 6b and Figure S6 in SI. Compared with the other non-precious metal and metal-free ORR electrocatalysts reported for acidic media, N-HPCNPs-900 also exhibit excellent catalytic activity (Table S5 in SI). Moreover, the kinetic current density of N-HPCNPs-900 (Fig. 6c) derived from the K-L plot (the inset of Fig. 6b) is estimated to be 39.19 mA cm^−2^ at −0.1 V *vs*. SCE, which is close in value to that of the benchmark Pt/C catalyst (41.9 mA cm^−2^).

To evaluate the selectivity of the ORR (whether it goes through two-electron versus four-electron reduction) in 0.5 M aqueous H_2_SO_4_ electrolyte, RRDE experiments over the materials are performed^45^ (Figure S5b in SI). The yield of H_2_O_2_ formed over N-HPCNPs-900 is about 7% in the potential range of −0.1 to +0.2 V *vs.* SCE, and the electron transfer number is calculated to be 3.86 (Fig. 6d). These results suggest that N-HPCNPs-900 strongly favor a four-electron ORR process.

**Figure 7:**
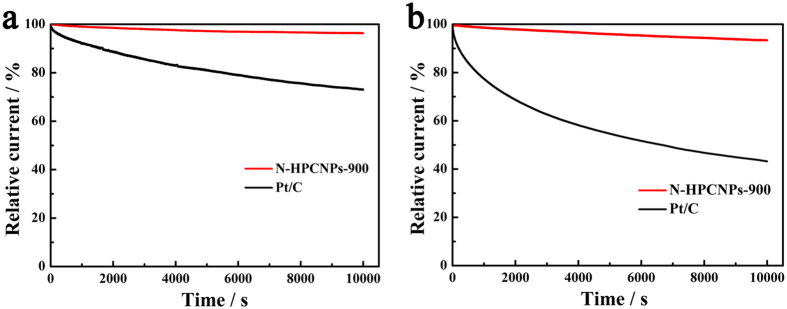
Evaluation of stability of N-HPCNPs-900 and Pt/C for 10,000 s in an O_2_-saturated electrolyte with a scan rate of 10 mV s^−1^ and rotation speed of 1600 rpm: (**a**) in 0.1 M KOH solution at −0.3 V *vs.* SCE and (**b**) in 0.5 M H_2_SO_4_ solution at 0.3 V *vs.* SCE.

In addition to the activity, the stability of electrocatalysts is another key parameter whether they can find practical applications as high-performance ORR catalysts in renewable energy systems. So, chronoamperometric experiments are conducted to investigate the electrochemical stability of N-HPCNPs-900 during ORR in both acid and alkaline media saturated with O_2_ (Fig. 7). While the benchmark electrocatalyst Pt/C electrode gives only about 73% and 42% of the initial current in 0.1 M aqueous KOH and 0.5 M aqueous H_2_SO_4_ solutions, respectively, after 10,000 s of ORR, that of N-HPCNPs-900 undergoes a current loss of only less than 6% in both KOH and H_2_SO_4_ media for the reaction in the same time period. This clearly indicates that N-HPCNPs-900 can serve as stable, practical electrocatalysts in lieu of the unstable and expensive Pt/C catalysts commonly used in conventional fuel cells^46^. Besides, when the ORR electrocatalysis over N-HPCNPs-900 is subjected to methanol by injecting 3 M methanol into O_2_-saturated acidic or alkaline solution (0.5 M aqueous H_2_SO_4_ or 0.1 M aqueous KOH solution) (Figure S7 in SI), no obvious decrease in current density is observed; however, in stark contrast to this, the commercial Pt/C catalyst shows a sharp decrease in current density. This indicates N-HPCNPs-900’s higher operational durability and better methanol tolerance than the benchmark Pt/C electrocatalyst.

The excellent electrocatalytic performance of N-HPCNPs-900 toward ORR can be attributed to the following aspects. First, the super-high surface area of the N-HPCNPs-900 helps creating more surface-exposed catalytically active sites that can promote ORR over the material^14^. Besides, the unique micro-/meso-porous structures and the high surface area-to-volume ratios in the material can lead to higher density of catalytic active sites and favorable structures that enable fast transport of reactants, ions, and electrons^14^,^34^,^35^,^36^,^47^. Additionally, the N-HPCNPs-900 possess the most desirable, catalytically active nitrogen-doped species for ORR (*i.e*., pyridinic-N and graphitic-N)^21^. Last, but not least, the metal-free texture and the high degree of graphitization (low *I*_D_/*I*_G_) in the material can contribute substantially to the catalytic activity of the hybrid catalyst for ORR and its excellent stability^21^.

## Conclusions

In summary, novel nitrogen-doped highly porous carbon nanoplatelets have been successfully synthesized from oatmeal (or a natural precursor rich with N and C) *via* a facile one-step pyrolysis method. The materials have been found to have catalytically beneficial features including super-high surface areas and hierarchical pore structures, which can give rise to more surface-exposed and accessible catalytic active sites in the materials, and thus high electrocatalytic activity toward ORR. For example, in alkaline medium, the N-HPCNPs-900 have exhibited excellent catalytic activity and good operational stability, superior to the benchmark Pt/C catalyst. Furthermore, the materials have presented comparable ORR catalytic performances as the commercial Pt/C in an acidic medium. Given the low-cost and availability of their precursors as well as their excellent electrocatalytic activity, outstanding durability and great stability against methanol crossover effect, the N-HPCNPs-900 hybrid materials can be very promising candidates to possibly replace the costly Pt-based electrocatalysts in electrochemical energy conversion devices.

## Methods

### Material synthesis

Nitrogen-doped highly porous carbon nanoplatelets (N-HPCNPs) were synthesized by a one-step pyrolysis method and an acid leaching procedure (Scheme 1). In a typical procedure, 300 mg of zinc acetate and 300 mg of urea were added into 5.0 mL of 1 M ferric trichloride aqueous solution with stirring, forming a homogeneous solution. Then, 200 mg of oatmeal was added into the above solution under continuous stirring. The solution was stirred for 24 h and then dried at 80 °C in an oven. The resulting dark brown product was obtained. The produced precursor was pyrolyzed at different temperatures (700, 800, 900, and 1000 °C) for an hour under a flow of Ar with the heating rate of 5 °C min^−1^, followed by acid leaching with 1 M aqueous HCl solution at room temperature for 24 h. The resulting hybrid carbon materials were denoted as N-HPCNPs-700, N-HPCNPs-800, N-HPCNPs-900 and N-HPCNPs-1000, respectively.

For comparison, control materials, namely N-P1CNPs, N-P2CNPs and PCNPs were synthesized by similar procedures as the one used to make N-HPCNPs-900 above. Specifically, they were synthesized without using zinc acetate, ferric trichloride or urea, respectively, under otherwise similar procedure as the one used to produce N-HPCNPs-900 above. Additionally, another control material, CNPs hybrid material was synthesized from the pristine oatmeal by pyrolyzed the oatmeal at 900 °C under an Ar atmosphere.

### Materials Characterization

Scanning electron microscopy (SEM, JSM-6701F, operating at 10 kV) and transmission electron microscopy (TEM, JEOL-2010, operating at 200 kV) were carried out to characterize morphologies and structures of the samples. Powder X-ray diffraction (XRD) measurements were performed on a RIGAK X-ray diffractometer (D/MAX2550 VB/PC, Japan) using a Cu Kα radiation (1.5418 Å) at a voltage of 40 kV. Raman spectra were measured on a Tri Vista^TM^ 555CRS Raman spectrometer at 532 nm of excitation. X-ray photoelectron spectroscopy (XPS) measurements were operated with an ESCLAB 250 spectrometer using X-ray source of Al Kα (1486.6 eV photons). The specific surface area determination and pore size distribution analysis were carried out by the Brunauer-Emmett-Teller (BET) method and density functional theory (DFT) pore model, respectively, using an ASAP2020 volumetric adsorption analyzer (Micromeritics) at 77 K.

### Preparation of the modified electrodes and electrocatalytic measurements

The working electrode (glassy carbon (GC) electrode) was first polished successively with 0.3 and 0.05 mm aluminum oxide slurries, and then thoroughly rinsed with distilled water, absolute ethanol and distilled water in turn for 1 min. After that, the cleaned glassy carbon (GC) electrode was blow-dried with N_2_ at ambient temperature. To modify the GC electrode, 1.0 mg N-HPCNPs-900 and 1.0 mL ethanol were mixed ultrasonically to obtain a homogeneous ink containing the catalyst, with a concentration of 1.0 mg mL^−1^. Following, a certain amount of catalyst ink was pipetted onto the GC electrode and the coated electrode was then left to dry in the air. After evaporation of the ethanol, 1 μL of a diluted Nafion solution (0.5 wt% Nafion) was put on top of the modified film, and then dried in the air. Finally, the total catalyst loading per area was calculated to be 0.4 mg cm^−2^. As comparison, the same amount of N-P1CNPs, N-P2CNPs, N-HPCNPs-b, PCNPs, CNPs and Pt/C (20 wt%) catalysts were also loaded onto the GC electrode by the same procedures above.

Electrochemical tests including rotating disk electrode (RDE), rotating ring-disk electrode (RRDE) and current–time (*i–t*) chronoamperometric response measurements were conducted using a computer-controlled potentiostat (CHI660E electrochemical workstation, CH Instrument, USA) with a three-electrode cell, in which the catalyst-modified GC electrode was employed as the working electrode, a saturated calomel electrode (SCE) was used as the reference electrode, and a platinum-wire electrode was employed as the auxiliary electrode. The ORR measurements involving RDE (5 mm in diameter) were carried out using a GC rotating-disk electrode with a scan rate of 10 mV s^−1^ in an O_2_-saturated 0.1 M aqueous KOH or 0.5 M aqueous H_2_SO_4_ solution. The ORR measurements involving RRDE (5.61 mm in diameter) were conducted using a GC rotating ring-disk electrode at a scan rate of 10 mV s^−1^ in an O_2_-saturated 0.1 M KOH or 0.5 M H_2_SO_4_ electrolyte. The collection efficiency of the platinum ring was 37%. In this work, to eliminate the possible influence of background non-Faradic current on potential shift, all cyclic voltammetry (CV) and linear sweep voltammetry (LSV) curves obtained in an O_2_-saturated electrolyte were based on the recordings obtained after background subtraction (i.e., by subtracting the CV or LSV currents obtained in N_2_-saturated electrolyte from the respective original curves obtained in O_2_-saturated solutions).

## Additional Information

**How to cite this article**: Zhang, Y. *et al*. A Facile Synthesis of Nitrogen-Doped Highly Porous Carbon Nanoplatelets: Efficient Catalysts for Oxygen Electroreduction. *Sci. Rep.* **7**, 43366; doi: 10.1038/srep43366 (2017).

**Publisher's note:** Springer Nature remains neutral with regard to jurisdictional claims in published maps and institutional affiliations.

## Supplementary Material

Supplementary Information

## Figures and Tables

**Figure 1:**
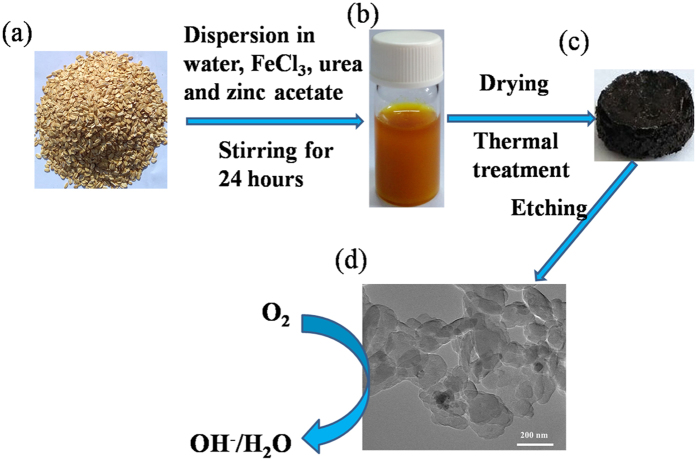
Schematic illustration of the procedures used to synthesize N-HPCNPs. (**a**) A photograph of oatmeal. (**b**) Stable suspension of oatmeal, urea and zinc acetate dispersed in a 1 M aqueous ferric chloride solution. (**c**) The bulk hybrid material obtained after thermal treatment and before acid etching (or N-HPCNPs-b). (**d**) TEM image of N-HPCNPs-900, the material pyrolyzed at 900 °C and then etched with 1 M HCl.

**Table 1:** Pore characteristics of N-HPCNPs-900, N-HPCNPs-b and CNPs.

Sample	Surface area/m^2^ g^−1^	Pore volume/cm^3^ g^−1^			
		Total pore	Micropore	Mesopore	Average pore diameter/nm
CNPs	984	0.55	0.32	0.18	3.7
N-HPCNPs-b	669	0.50	0.12	0.34	3.7
N-HPCNPs-900	2633	1.78	0.52	1.11	3.9

Total pore obtained at P/P_0_ = 0.99. Micropore and mesopore are determined by DFT method. Average pore diameter is determined by BJH method.
