# Supplementary material for: A Facile Synthesis of Nitrogen-Doped Highly Porous Carbon Nanoplatelets: Efficient Catalysts for Oxygen Electroreduction
Source: Sci Rep. 2017 Feb 27;7:43366. doi: 10.1038/srep43366 (PMC5327468; doi:10.1038/srep43366)
Supplement: Supplementary Information [file srep43366-s1.pdf]

Supplementary Information for

A Facile Synthesis of Nitrogen-Doped Highly Porous Carbon Nanoplatelets: Efficient Catalysts for Oxygen Electroreduction

Yaqing Zhang[1], Xianlei Zhang[1], Xiuxiu Ma[1], Wenhui Guo[1], Chunchi Wang[1], Tewodros Asefa[2*] and Xingquan He[1*]

[1] *Department of Chemistry and Chemical Engineering, Changchun University of Science and Technology, Changchun 130022, P. R. China.*

[2] *Department of Chemistry and Chemical Biology & Department of Chemical and Biochemical Engineering, Rutgers, The State University of New Jersey, Piscataway, NJ 08854, United States.*

.

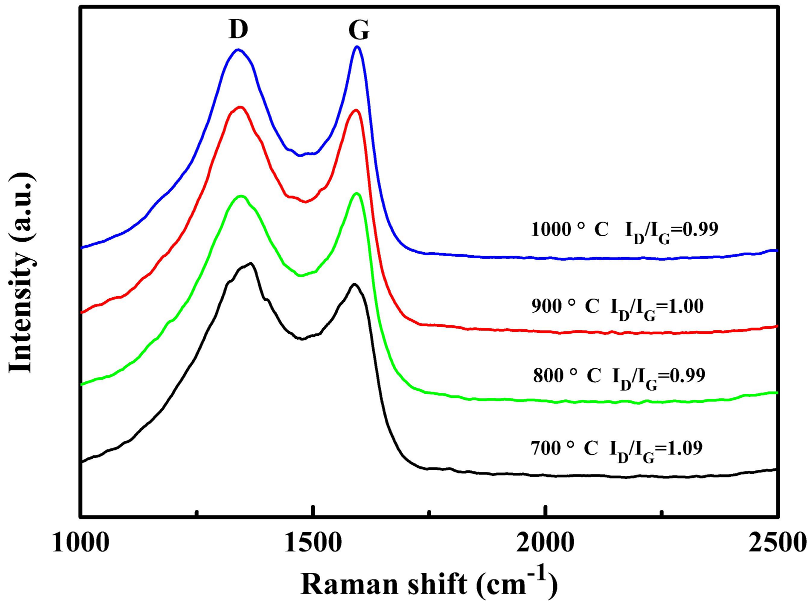

Figure S1. Raman spectra of N-HPCNPs obtained at different pyrolysis temperatures.

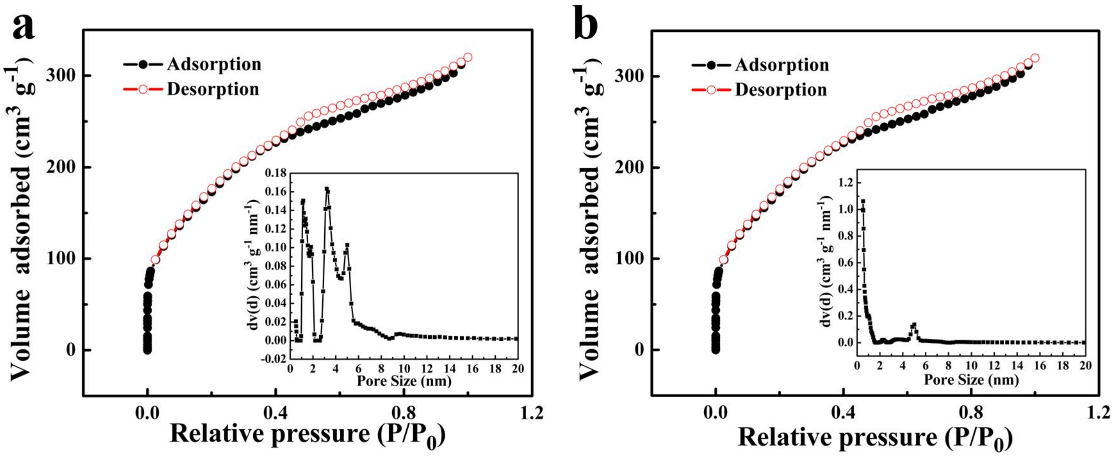

Figure S2. Nitrogen adsorption-desorption isotherms of N-HPCNPs-b a) and CNPs b). The inset shows the pore size distribution obtained by using the DFT method.

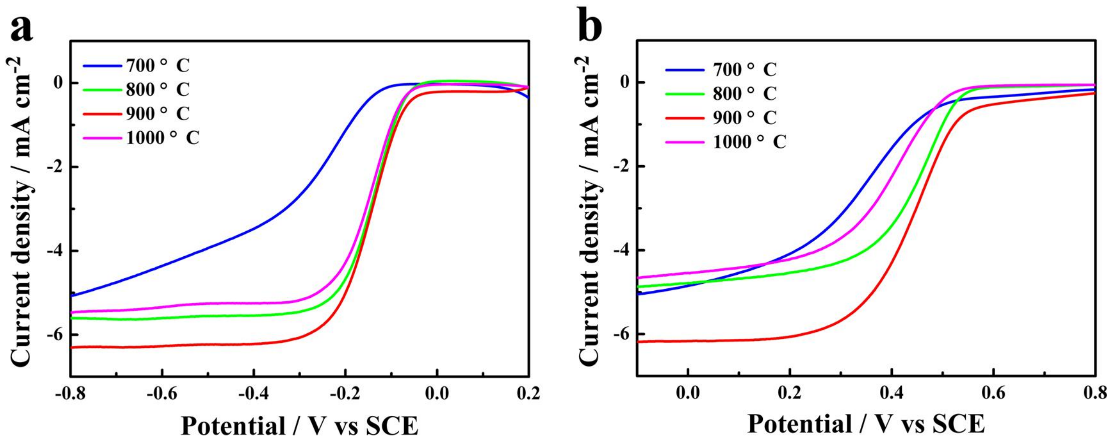

Figure S3. The background-corrected RDE polarization curves of the N-HPCNPs catalysts pyrolyzed at different temperatures in an O_2-saturated electrolyte with scan rate of 10 mV s^{-1} and rotation speed of 1600 rpm: a) in 0.1 M aqueous KOH and b) in 0.5 M aqueous H_2SO_4 solutions.

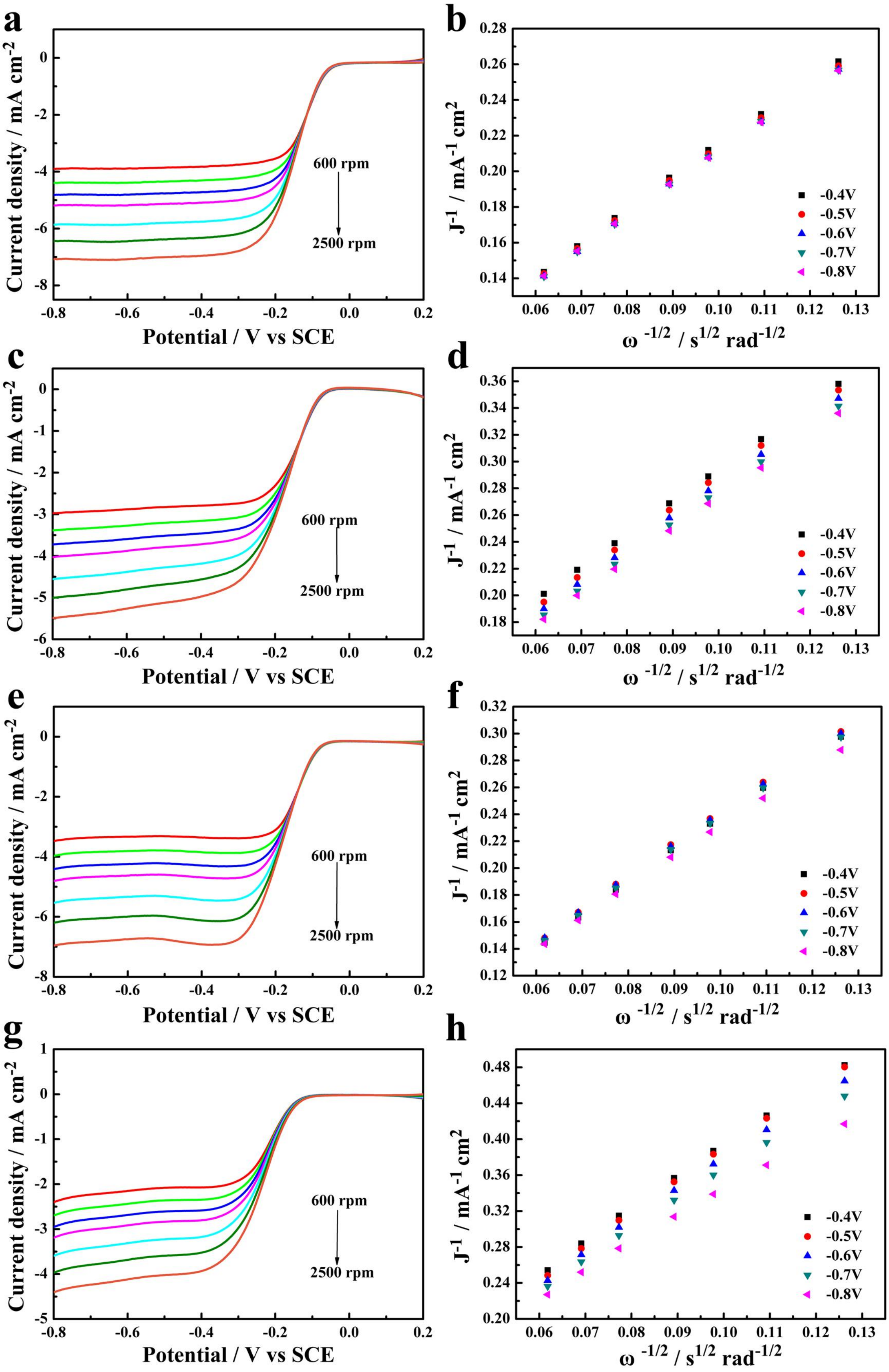
a
b
c
d
e
f
g
h
Current density / mA cm-2
Potential / V vs SCE
600 rpm
2500 rpm
J-1 / mA-1 cm2
ω -1/2 / s1/2 rad-1/2
-0.4V
-0.5V
-0.6V
-0.7V
-0.8V

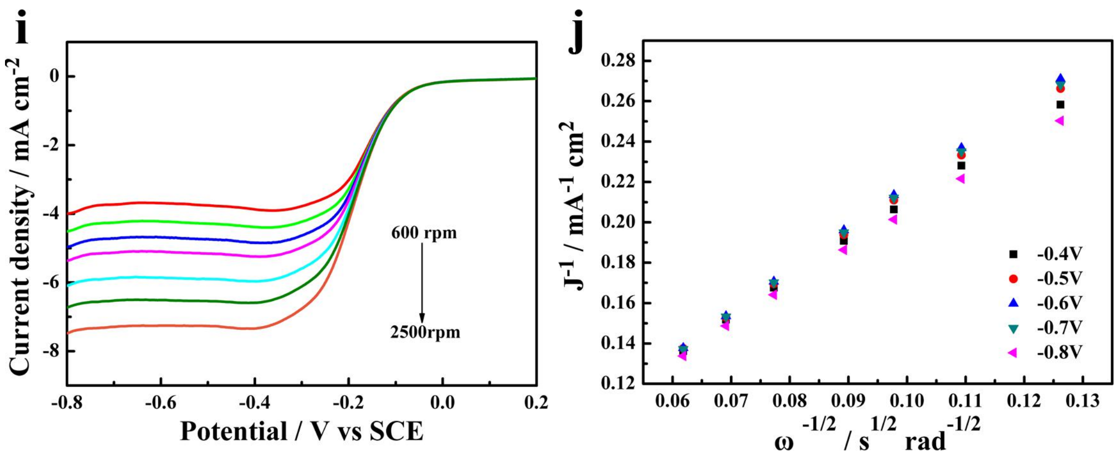

Figure S4. (a-j) Background-corrected LSV curves and corresponding K-L plots of different materials synthesized. Background-corrected LSV curves of a) N-P1CNPs, c) N-P2CNPs, e) PCNPs, g) CNPs and i) Pt/C at different rotation speeds in an O_2-saturated 0.1 M aqueous KOH solution with the scan rate of 10 mV s^{-1}. The corresponding K-L plots of b) N-P1CNPs, d) N-P2CNPs, f) PCNPs, h) CNPs, and j) Pt/C at potentials of -0.4, -0.5, -0.6, -0.7 and -0.8 V *vs.* SCE.

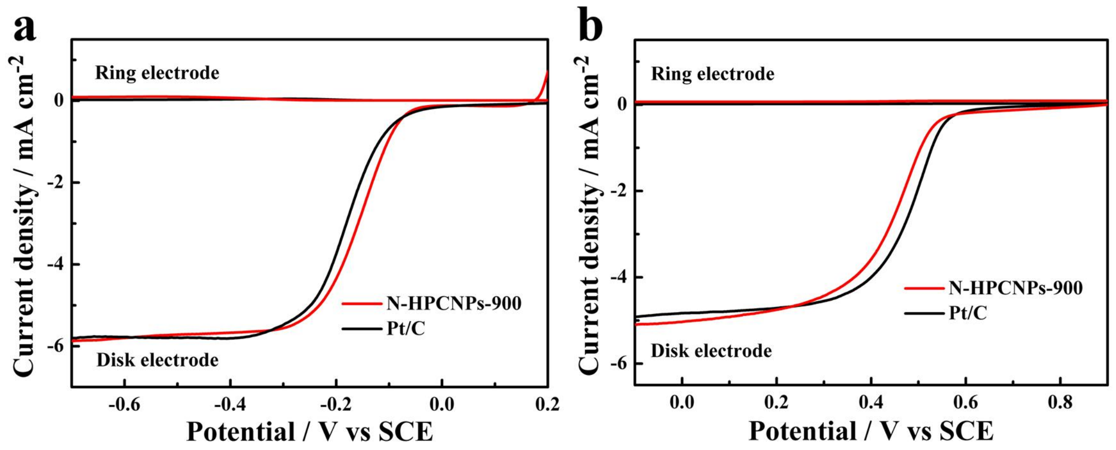

Figure S5. Background-corrected RRDE linear sweep voltammograms of N-HPCNPs-900 and Pt/C in an O_2-saturated electrolyte with the scan rate of 10 mV s^{-1} and rotation speed of 1600 rpm: a) in 0.1 M aqueous KOH and b) in 0.5 M aqueous H_2SO_4 solutions.

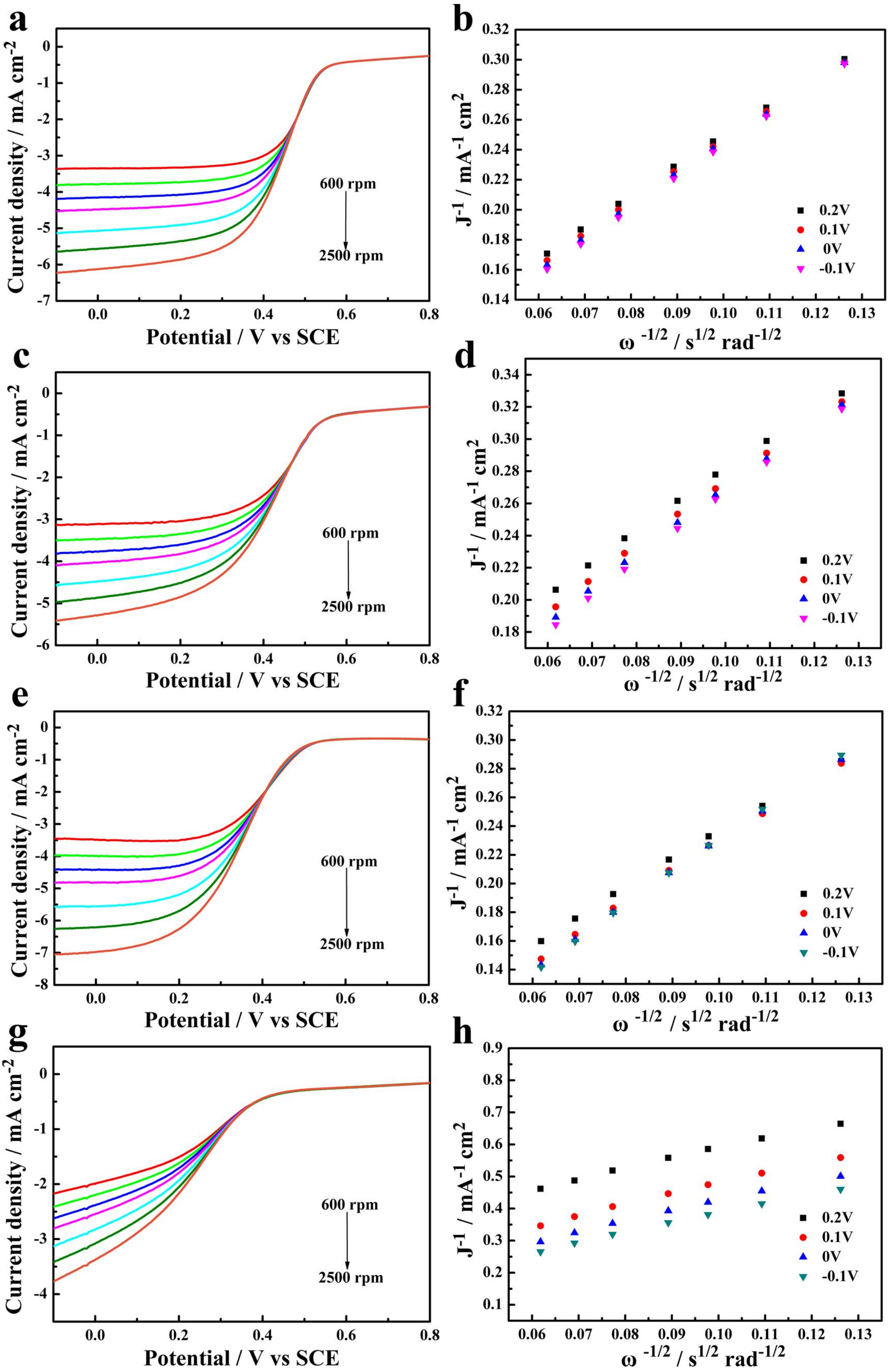
a
Current density / mA cm^{-2}
Potential / V vs SCE
600 rpm
2500 rpm
b
J^{-1} / mA^{-1} cm^{2}
$\omega^{-1/2}$ / s$^{1/2}$ rad$^{-1/2}$
0.2V
0.1V
0V
-0.1V
c
Current density / mA cm^{-2}
Potential / V vs SCE
600 rpm
2500 rpm
d
J^{-1} / mA^{-1} cm^{2}
$\omega^{-1/2}$ / s$^{1/2}$ rad$^{-1/2}$
0.2V
0.1V
0V
-0.1V
e
Current density / mA cm^{-2}
Potential / V vs SCE
600 rpm
2500 rpm
f
J^{-1} / mA^{-1} cm^{2}
$\omega^{-1/2}$ / s$^{1/2}$ rad$^{-1/2}$
0.2V
0.1V
0V
-0.1V
g
Current density / mA cm^{-2}
Potential / V vs SCE
600 rpm
2500 rpm
h
J^{-1} / mA^{-1} cm^{2}
$\omega^{-1/2}$ / s$^{1/2}$ rad$^{-1/2}$
0.2V
0.1V
0V
-0.1V

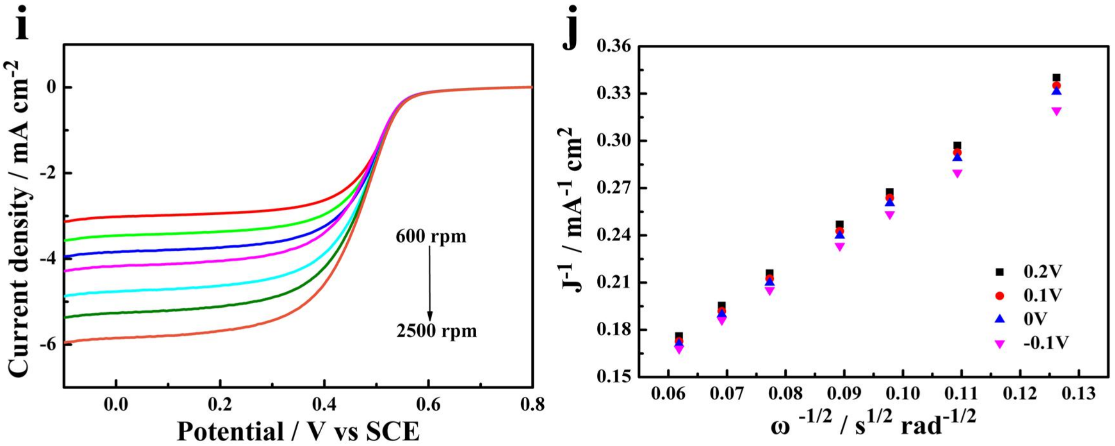

Figure S6. Background-corrected LSV curves and corresponding K-L plots of different materials synthesized. Background-corrected LSV curves of a) N-P1CNPs, c) N-P2CNPs, e) PCNPs, g) CNPs and i) Pt/C at different rotation speeds in an O_2-saturated 0.5 M aqueous H_2SO_4 with the scan rate of 10 mV s^{-1}. K-L plots of b) N-P1CNPs, d) N-P2CNPs, f) PCNPs, h) CNPs, and j) Pt/C at fixed potentials of 0.2, 0.1, 0 and -0.1 V *vs.* SCE.

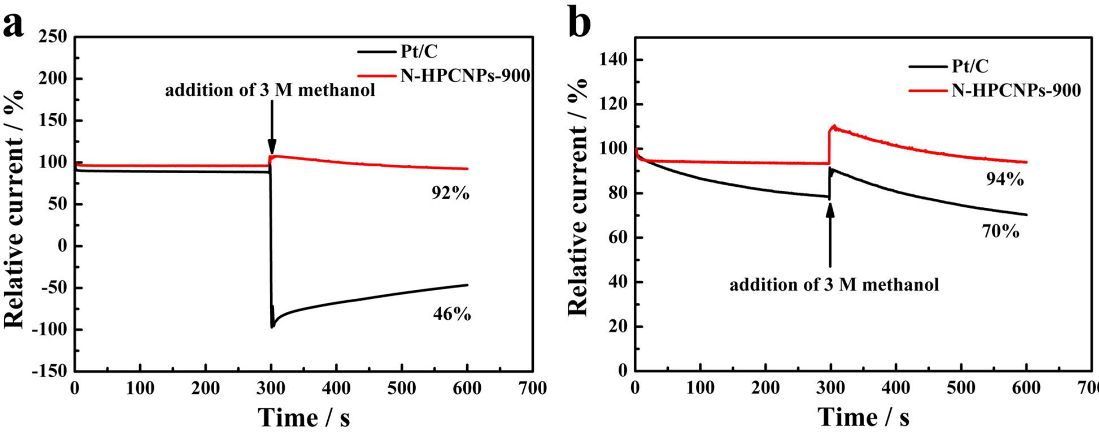

Figure S7. Current density time chronoamperometric responses of Pt/C and N-HPCNPs-900 in an O_2-saturated electrolyte with the scan rate of 10 mV s^{-1} and rotation speed of 1600 rpm: a) in 0.1 M aqueous KOH and b) in 0.5 M aqueous H_2SO_4 solutions. The arrow indicates the time at which methanol is added into the electrolytic cell.

Sample	Surface area ($m^2\ g^{-1}$)	Pore volume ($cm^3\ g^{-1}$)	Average pore diameter (nm)	References
N-HPCNPs-900	2633	1.78	3.9	In this work
NCMS	995	0.50	<2	1
NC-A	2191	--	2.6	2
SS-AW	390	0.69	6.89	3
R-3DNG	549	1.76	--	4
BP-800	1578	1.09	<2	5
Co, N-CNF	1170	1.52	--	6
N-MCNs-7-900	1117	1.77	98	7
HPGC	970	0.69	2.85	8
NGPC-1000-10	932	0.99	--	9
Fe-N-CNFs	425	0.44	6.85	10
FeCo-OMPC	1190	1.40	4.85	11
N,B-GA-1000	546	1.21	88.3	12
TTF-F	2570	2.14	3.33	13
CNTHb-700	459	--	3.75	14
Fe-P-900	1371	0.75	--	15

Table S1. Summary of the porous structural features of some relevant carbon materials reported in literature compared with the one reported herein.

Electrocatalysts	E_{onset} V *vs.* SCE	$E_{1/2}$ V *vs.* SCE	j_L (mA cm^{-2}) at -0.8 V *vs.* SCE	j_K (mA cm^{-2}) at -0.5 V *vs.* SCE
N-HPCNPs-900	-0.018	-0.148	6.50	59.95
N-P1CNPs	-0.018	-0.138	5.86	31.20
N-P2CNPs	-0.043	-0.173	4.55	22.89
PCNPs	-0.042	-0.166	5.54	50.92
CNPs	-0.088	-0.238	3.59	20.31
Pt/C	-0.020	-0.181	6.09	48.13

Table S2. Electrochemical parameters in the measurement of ORR, estimated from RDE polarization curves in an O_2-saturated 0.1 M KOH electrolyte (obtained from Fig. 5b).

Electrocatalyst	ΔE_{onset}[a,c] (V)	$\Delta E_{1/2}$[a,c] (V)	j_L[b,c] [mA cm^{-2}]	Catalyst loading Per area[μg cm^{-2}]	Reference electrode	Ref.
N-HPCNPs-900	0	0.03	6.5	400	SCE	In this work
NG-C	-0.04	-0.05	5.7	Not mentioned	Ag/AgCl	16
NHPCM-1000	-0.1	-0.07	5.79	320	RHE	17
N-C@CNT-900	-0.03	-0.05	4.7	400	RHE	18
N,P-CGHNs	-0.03	0.01	5.6	300	RHE	19
B,N-graphene	-0.11	-0.13	5.2	280	RHE	20
N-S-CMK-3	-0.05	-0.03	5.9	306	RHE	21
LDH@ZIF-67-800	-0.03	0.02	5.5	200	RHE	22
TTF-700-96	-0.14	-0.07	5.0	300	RHE	13
Fe_3C/NG-800	0.06	0.05	6.0	400	RHE	23

Table S3. The comparison of the ORR performance of different catalysts in 0.1 M KOH electrolyte. [a] Represents the difference in onset potential or half-wave potential between the various catalysts and Pt/C. [b] Represents the diffusion-limited current density of the various catalysts at a rotation speed of 1600 rpm. [c] The onset potential (E_{onset}), half-wave potential ($E_{1/2}$) and diffusion limited current density (j_L) were obtained from the corresponding literatures and the corresponding figures in the present study.

Electrocatalysts	E_{onset} V *vs.* SCE	$E_{1/2}$ V *vs.* SCE	j_L (mA cm^{-2}) at -0.1 V *vs.* SCE	j_K (mA cm^{-2}) at -0.1 V *vs.* SCE
N-HPCNPs-900	0.588	0.445	6.18	39.19
N-P1CNPs	0.588	0.460	5.13	32.86
N-P2CNPs	0.588	0.435	4.57	17.52
PCNPs	0.572	0.375	5.57	52.12
CNPs	0.484	0.247	3.13	11.88
Pt/C	0.601	0.474	4.87	41.86

Table S4. Electrochemical parameters for ORR estimated from RDE polarization curves in 0.5 M H_2SO_4 electrolyte (obtained from Fig. 6a).

Electrocatalysts	E_{onset}[a] (V)	$E_{1/2}$[a] (V)	j_L [a][mA cm^{-2}]	Media	Catalyst loading Per area[μg cm^{-2}]	Reference electrolyte	Ref.
N-HPCNPs-900	0.59	0.45	6.18	0.5M H_2SO_4	400	SCE	In this work
N-C@CNT-900	0.81	0.60	3.79	0.5M H_2SO_4	400	RHE	18
N-CNTs	0.65	0.45	2.0	0.5M H_2SO_4	Not mentioned	RHE	24
N-doped graphene	0.68	0.15	2.0	0.5M H_2SO_4	50	RHE	25
Fe-N-CNF	0.84	0.62	5.0	0.5M H_2SO_4	600	RHE	10
N-carbon spheres	0.65	0.42	5.5	0.5M H_2SO_4	250	RHE	26
Fe-N-C	0.82	0.6	6	0.1M $HClO_4$	100	RHE	27
N,P-CGHNs	0.9	0.68	5.7	0.1M $HClO_4$	600	RHE	19
Fe_3C/NG-800	0.92	0.77	6.2	0.1M $HClO_4$	400	RHE	23
LDH@ZIF-67-800	0.875	0.675	5.1	0.1M $HClO_4$	200	RHE	22

Table S5. Comparison of the performance of different catalysts for ORR in 0.5 M H_2SO_4 electrolyte. [a] The onset potential (E_{onset}), half-wave potential ($E_{1/2}$) and diffusion limited current density (j_L) were obtained from the corresponding literatures and the corresponding figures in present study.

References for Supporting Information

1. Kim, S. Y. *et al*. Template-free synthesis of high surface area nitrogen-rich carbon microporous spheres and their hydrogen uptake capacity. *J. Mater. Chem. A* **2,** 2227-2232 (2014).
2. He, W. H., Jiang, C. H., Wang, J. B. & Lu, L. H. High-rate oxygen electroreduction

over graphitic-N species exposed on 3D hierarchically porous nitrogen-doped carbons. *Angew. Chem., Int. Ed.* **126,** 9657-9661 (2014).

3. Yuan, S.-J. & Dai, X.-H. Facile synthesis of sewage sludge-derived in-situ multi-doped nanoporous carbon material for electrocatalytic oxygen reduction. *Sci. Rep.* **6,** 27570; 10.1038/srep27570 (2016).
4. Qin, Y. *et al*. Crosslinking graphene oxide into robust 3D porous N-doped grapheme. *Adv. Mater.* **27,** 5171-5175 (2015).
5. Zhu, H., Yin, J., Wang, X. L., Wang, H. Y. & Yang, X. R. Microorganism-derived heteroatom-doped carbon materials for oxygen reduction and supercapacitors. *Adv. Funct. Mater.* **23,** 1305-1312 (2013).
6. Shang, L. *et al*. Well-dispersed ZIF-derived Co,N-Co-doped carbon nanoframes through mesoporous-silica-protected. *Adv. Mater.* **28,** 1668-1674 (2016).
7. Wang, G. *et al*. Controlled synthesis of N-doped carbon nanospheres with tailored mesopores through self-assembly of colloidal silica. *Angew. Chem., Int. Ed.* **54,** 15191-15196 (2015).
8. Wang, D. W., Li, F., Liu, M., Lu, G. Q. & Cheng, H. M. 3D aperiodic hierarchical porous graphitic carbon material for high-rate electrochemical capacitive energy storage. *Angew. Chem., Int. Ed.* **47,** 373-376 (2008).
9. Zhang, L. J. *et al*. Highly graphitized nitrogen-doped porous carbon nanopolyhedra derived from ZIF-8 nanocrystals as efficient electrocatalysts for oxygen reduction reactions. *Nanoscale* **6,** 6590-6602 (2014).
10. Wu, Z. Y. *et al*. Iron carbide nanoparticles encapsulated in mesoporous Fe-N-doped

carbon nanofibers for efficient electrocatalysis. *Angew. Chem., Int. Ed.* **54,** 8179-8183 (2015).

11. Cheon, J. Y. *et al.* Ordered mesoporous porphyrinic carbons with very high electrocatalytic activity for the oxygen reduction reaction. *Sci. Rep.* **3,** 2715; 10.1038/srep02715 (2013).
12. Xu, C. C., Su, Y., Liu, D. J. & He, X. Q. Three-dimensional N,B-doped graphene aerogel as a synergistically enhanced metal-free catalyst for the oxygen reduction reaction. *Phys. Chem. Chem. Phys.* **17,** 25440-25448 (2015).
13. Hao, L. *et al.* Bottom-up construction of triazine-based frameworks as metal-free electrocatalysts for oxygen reduction reaction. *Adv. Mater.* **27,** 3190-3195 (2015).
14. Vij, V., Tiwari, J. N., Lee, W.-G., Yoon, T. & Kim, K. S. Hemoglobin-carbon nanotube derived noble-metal-free Fe_5C_2-based catalyst for highly efficient oxygen reduction reaction. *Sci. Rep.* **6,** 20132; 10.1038/srep20132 (2016).
15. Singh, K. P., Bae, E. J. & Yu, J.-S. Fe-P: A new class of electroactive catalyst for oxygen reduction reaction. *J. Am. Chem. Soc.* **137,** 3165-3168 (2015).
16. Liao, Y. L. *et al.* Facile fabrication of N-doped graphene as efficient electrocatalyst for oxygen reduction reaction. *ACS Appl. Mater. Interfaces* **7,** 19619-19625 (2015).
17. Kibsgaard, J., Chen, Z., Reneicke, B. N. & Jaramillo, T. F. An in situ source-template-interface reaction route to 3D nitrogen-doped hierarchical porous carbon as oxygen reduction electrocatalyst. *Nat. Mater.* **11,** 963-969 (2012).
18. Guo, C. Z., Liao, W. L., Li, Z. B., Sun, L. T. & Chen, C. G. Easy conversion of protein-rich enoki mushroom biomass to a nitrogen-doped carbon nanomaterial as a

promising metal-free catalyst for oxygen reduction reaction. *Nanoscale* **7,** 15990-15998 (2015).

19. Yang, J. *et al.* A highly efficient metal-free oxygen reduction electrocatalyst assembled from carbon nanotubes and graphene. *Adv. Mater*. **28,** 4606-4613 (2016).
20. Zhen, Y., Jiao, Y., Ge, L., Jaroniec, M. & Qiao, S. Z. Two-step boron and nitrogen doping in graphene for enhanced synergistic catalysis. *Angew. Chem., Int. Ed*. **52,** 3110-3116 (2013).
21. Qiu, Y., Huo, J. J., Jia, F., Shanks, B. H. & Li, W. Z. N- and S-doped mesoporous carbon as metal-free cathode catalysts for direct biorenewable alcohol fuel cells. *J. Mater. Chem. A* **4,** 83-95 (2016).
22. Li, Z. H. *et al.* Directed growth of metal-organic frameworks and their derived carbon-based network for efficient electrocatalytic oxygen reduction. *Adv. Mater.* **28,** 2337-2344 (2016).
23. Xiao, M. L., Zhu, J. B., Feng, L. G., Liu, C. P. & Xing, W. Meso/macroporous nitrogen-doped carbon architectures with iron carbide encapsulated in graphitic layers as an efficient and robust catalyst for the oxygen reduction reaction in both acidic and alkaline solutions. *Adv. Mater*. **27,** 2521-2527 (2015).
24. Yu, D. S., Zhang, Q. & Dai, L. M. Highly efficient metal-free growth of nitrogen-doped single-walled carbon nanotubes on plasma-etched substrates for oxygen reduction. *J. Am. Chem. Soc*. **132,** 15127-15129 (2010).
25. Parvez, K. *et al.* Nitrogen-doped graphene and its iron-based composite as efficient

electrocatalysts for oxygen reduction reaction. *ACS Nano* **6,** 9541-9550 (2012).

26. Ai, K. L., Liu, Y. L., Ruan, C. P., Lu, L. H. & Lu, G. Q. Sp^2 C-dominant N-doped carbon sub-micrometer spheres with a tunable size: A versatile platform for highly efficient oxygen-reduction catalysts. *Adv. Mater*. **25,** 998-1003 (2013).
27. Lin, L., Zhu, Q. & Xu, A. W. Noble-metal-free Fe-N/C catalyst for highly efficient oxygen reduction reaction under both alkaline and acidic conditions. *J. Am. Chem. Soc*. **136,** 11027-11033 (2014).
